# You Only Live Once! Understanding Indonesian and Taiwan Travel Intention During COVID-19 Pandemic

**DOI:** 10.3389/fpsyg.2022.922529

**Published:** 2022-09-02

**Authors:** Lusy Asa Akhrani, Wen Cheng, Ika Herani, Yuyun Agus Riani, Resti Diah Pratiwi, Aqsha Ade Fahmi, Aubrey Ammaritza, M. Haikal Azaim Barlamana

**Affiliations:** ^1^Department of Psychology, Universitas Brawijaya, Malang, Indonesia; ^2^Institute of Education Development, Center for Teacher Education, National Sun Yat-sen University, Kaohsiung, Taiwan

**Keywords:** COVID-19 risk perception, fear of COVID-19, fear to travel, Indonesia, risk perception to travel, Taiwan vaccine attitude

## Abstract

Indonesia and Taiwan are two countries that have been affected by the tourism sector, although with different policies to control the COVID-19 pandemic. Taiwan is known as a country with pandemic policies that have been recognized around the world, although it has a high vulnerability to experiencing a high number of infections due to its geographical and political position close to the source of the pandemic. On the other hand, Indonesia is known for its controversial pandemic management and control policies. Indonesia and Taiwan have carried out various public policies to increase tourism activities during the pandemic, such as accelerating vaccination in tourist areas and for tourists, as well as various other stimuli to stimulate tourism. The debate over vaccination raises questions about attitudes toward vaccines in society. The lack of clarity on psychosocial and political conditions creates confusion among the public in perceiving the COVID-19 pandemic and in perceiving the risks of traveling. This can affect people's attitudes toward vaccines, travel anxiety, and travel intentions. This study aims to analyze traveling intention due to the COVID-19 pandemic through COVID-19 Risk Perception, Fear of COVID-19, Risk Perception to Travel, Vaccine Attitude, and Fear to Travel. The research in Indonesia involved 358 respondents while the research in Taiwan involved 283 respondents. The research analysis used multiple regression and simple linear regression to ascertain the role of each association. The results showed that the travel intention of Indonesian tourists was formed from the direct and indirect roles of covid 19 risk perception, fear of covid 19, risk perception to travel, vaccine attitude, and fear to travel. Meanwhile, the travel intention of Taiwanese tourists is not influenced by a fear of covid. The travel intention model of Taiwanese tourists is formed from the direct and indirect roles of covid 19 risk perception, risk perception to travel, vaccine attitude, and fear to travel. This research contributes to tourism risk management in the face of pandemics, particularly in terms of government policies that can reduce tourism anxiety to travel during disasters.

## Introduction

The COVID-19 pandemic has hit many sectors, one of which is the tourism sector. Several countries that rely on foreign exchange from the tourism industry have experienced a slump due to the pandemic. This sudden situation makes people unprepared to deal with it both physically and psychologically (Sabir and Phil, 2016). The COVID-19 pandemic has brought people into situations in which they experience psychological problems such as fear and anxiety about contracting the virus (Fitria, 2020; Muslim, 2020). Research conducted by Le and Nguyen (2021), found that the COVID-19 pandemic increases the anxiety, worry, and feelings of discomfort felt by individuals, which results in avoiding people (Taylor, 2019). Fear is one of the hallmarks of infectious diseases (Ahorsu et al., 2020), and has the potential to be a stressor that affects an individual's life (Muslim, 2020).

The decline in mental wellbeing during a pandemic due to feeling fear as a result of risk perception of COVID-19. In regard to tourism, Luo and Lam (2020) conducted research in Hong Kong which found that people were aware of safety when traveling, therefore fear of COVID-19 can affect travel anxiety and risk. Additionally, risk perception in tourism can be associated with the evaluation of the situation regarding the risk in deciding on a trip (traveling), buying or consuming products, as well as travel experiences (Reisinger and Mavondo, 2005), relating to the possibility of the action of a hazard that can affect travel decisions (Chew and Jahari, 2014). The findings of a study by Neuburger and Egger (2021) showed a rapid rise in COVID-19 risk perception, travel risk perception, and travel behavior. However, its perception could vary between countries, as Dryhurst et al. (2020) revealed that the perception of COVID-19 risk was consistently correlated with several experiential and socio-cultural factors in various countries.

According to Artuger (2015) and Wang et al. (2021), travel and tourism are more vulnerable to risk, therefore, travelers are more sensitive to safety issues and risks. Thus, it was suggested that tourists must postpone or cancel their plans to anticipate risks during the pandemic. Moreover, risk perception among tourist has four dimensions, namely financial risk, time risk, social-psychological risk, and health risk (Fauzan, 2020; Utama and Setiawan, 2020). In terms of tourist mobility, the country situations should also be considered because each country's policy in handling the pandemic differ. For instances, Taiwan is an island with a population of 23 million people, and it is known around the world for its successful response to the COVID-19 outbreak. In contrast, Indonesia, a country with more than a thousand islands, is attempting to rebuild its economy while dealing with the pandemic by implementing a number of controversial policies.

During the pandemic's containment attempts in Indonesia, a discrepancy arose with the Minister of Tourism and Creative Economy for the 2020 period, Sandiaga Uno, when he issued an innovation called Work from Destination. This innovation is a strategy for increasing revenue in the tourism industry by encouraging workers to work in tourist areas (Tempo, 2021). The arrival of employment from the destination can encourage people who have already traveled to continue traveling, or encourage people who have not traveled to travel, which contradicts the attempts to mitigate COVID-19 by decreasing social mobilizations. Similarly, COVID-19 spokeswoman Achmad Yurianto warned the public not to travel unless it is necessary, as travel increases the danger of coronavirus transmission. Moreover, the source of transmission from those who have no symptoms is difficult to detect, thus confirmed positive cases continue to grow (Coverage 6, 2020). In addition, the lack of public awareness is one of the factors for the rising positive cases (Yatimah et al., 2020).

While public awareness helps to prevent the spread of infectious diseases, many people in Indonesia continue to travel during the pandemic. The number of domestic tourists in Indonesia has surged by up to 96 percent during the pandemic, according to Traveloka founder Albert Zhang (Business News, 2020). For example, domestic visitors in Jakarta, Yogyakarta, and Bali also experienced an increase (Cahya, 2020; Damarjati, 2020; Saputra, 2020; Sugiari, 2021). In addition, in Indonesia some individuals comply with health protocols, but some ignore government regulations and mingle in public places (Cori et al., 2020). Moreover, Setiyawati (2020) stated that there are still many people who violate health protocols when doing activities outside the home as a form of despair about their conditions or situations because the impact of the COVID-19 pandemic has been so great on their lives (Damarjati, 2020; Sugiari, 2021).

Conversely, Taiwan has successfully managed the epidemic because of early implementation of strong border controls and a very efficient tracing mechanism. The country has reported 18,041 cases to date out of a population of 23.5 million (Nugrahani, 2022). Closing the departure or entry gates from the beginning of the pandemic prevented an increase in COVID-19 cases, as well as access to travel or cross-country travel. Furthermore, when positive cases occur, extremely tight prohibitions are implemented in public transportations, restaurants, schools, and other areas. Taiwanese stakeholders, including institutions and individuals, follow the COVID-19 health protocol very rigorously, for instance fines are actually enforced to those who violate.

One of the measures to overcome COVID-19 is the provision of vaccines. In Indonesia, though the method has resulted in various reactions and still has pros and cons, this vaccine is expected to be given to all Indonesian people with determined priority stages, health workers, community leaders, teachers, ministry/institutional apparatus, vulnerable communities, ultimately the community, and other economic actors (Minister of Health of the Republic of Indonesia, 2021). As it could reduce COVID-19 risk, vaccination is also a new hope for the tourism industry, which has been sluggish for more than a year. Therefore, some areas with a focus on foreign exchange from the tourism industry such as Bali are preparing to vaccinate residents and workers in the tourism sector.

While in Taiwan, the government has intensively forced vaccination, and has been generating vaccines for locals since the outbreak began. Although the conflict with China had prevented Taiwan from getting the vaccine at the beginning of the Pandemic, the Taiwanese government has so far succeeded in providing more than 70% of people in Taiwan with two doses of the vaccine, and currently, the government is launching booster injections, so far around 10% of the population has received the third injection (Nugrahani, 2022). The Taiwan Epidemic Command Center (CEEC) recommended that local governments provide each individual who wishes to be vaccinated a gift of 200 Taiwan dollars (about 102 thousand rupiahs) to optimize vaccine coverage and encourage Taiwanese citizens' enthusiasm in COVID-19 vaccination (Erina, 2022).

The way Indonesia and Taiwan handled the COVID-19 outbreak has become a fascinating case study in travel behavior as can be seen Table 1. It is appealing in comprehending the theoretical model of travel intentions between Indonesia and Taiwan, as the protective activities taken after recognizing a high health risk are determined by the mitigation techniques available to the individual. These two countries offered different techniques, for example the Indonesian government released several tourism stimuli however Taiwan's government executed a lockdown system. These two Asian countries, which have distinct characteristics in terms of geography and policy system, are worth investigation in relation to tourist concerns during the COVID-19 period, with a focus on health risks. Individual decision-making is influenced significantly by risk perceptions, therefore, we examine the differences between these countries by addressing questions such as whether COVID-19 risk perception, fear of COVID 19, risk perception to travel, vaccine attitude, and fear to travel as independent variables contribute to travel intention as dependent variables. We take into account that these five elements could influence travel intensity since different travel patterns generate different types of individual behavior, no research has been done to compare the tourist intensity between countries during COVID-19 pandemic. The present paper is divided into five sections, Section COVID-19 Risk Perception provides the data and methodology. Section Results describes the different test results of our hypothesis on how COVID-19 risk perception scenario contributes to travel intentions. Section Discussion presents the interpretation, rationale, and application of our findings to demonstrate the similarities and differences between Indonesian and Taiwanese. Last, Section Conclusion, Limitation, and Future Direction consists of conclusions, limitations, and recommendations for the future directions.

**Table 1:** Differences in handling the COVID-19 pandemic in Taiwan and Indonesia.

**Policy**	**Taiwan**	**Indonesia**
Lockdown	Taiwan has never imposed a strict lockdown. The government also does not impose very strict restrictions on citizens' freedom.	Indonesia rejects the term lockdown and replaces it with various terms such as regional quarantine, PSBB, MIkro PPKM, Macro PPKM, and others.
Pandemic response	Taiwan handling focus on speed	Unclear between Health and economy
	Taiwanese authorities began screening passengers on direct flights from Wuhan, where the virus was first identified.	There is no closure of Indonesia's entry and exit for several sectors such as foreign workers, diplomats and others. Flights from the country of origin of the virus are also not closed.
First Case Confirmation	Taiwan confirms first case of coronavirus on January 21	Indonesia only admitted the first case in March, the first 3 months (January to March) all state officials tried to deny the fact that the pandemic could enter Indonesia.
Close the entrance	Ban residents from Wuhan from visiting and entering Taiwan. All passengers arriving from mainland China, Hong Kong and Macau are required to undergo screening. Until March, Taiwan banned all foreign nationals from entering its territory, except for diplomats and those with special visas.	Entrances are open, closures are issued in policy but inflows at airports are still open.
Increase Outbreak Management Capacity	activate the Central Epidemic Command Center, which was built after SARS, for inter-ministerial coordination. The government has also increased the production of masks and protective equipment to ensure a stable domestic supply of PPE. Taiwan has firmly even banned the export of masks within weeks. This is to ensure that domestic stock is maintained.	Establishing a Task Force for Handling the Acceleration of COVID-19 (GTPP)
Contact Tracking and Quarantine	The government is also investing in rapid and effective mass testing and contact tracing. They are also carrying out coronavirus testing across the country, including re-testing people who previously had a history of pneumonia of unknown cause. Very careful contact tracing, and very strict close contact quarantine is the best way to contain COVID-19	Low contact tracing, low Covid test
information transparency	The Taiwanese government has always provided open and transparent information regarding the COVID-19 pandemic. The government also announced the latest pandemic situation and the source of each case, contacts, and the follow-up process (Pristiandaru, 2020).	Data is confusing, some cases show data that is not recorded
Smart app	Taiwan's Central Pandemic Command Headquarters uses mobile phones to track all passengers entering Taiwan and strictly enforces 14-day quarantine measures. In addition, the many applications that were developed for the public to check the stock of masks and alert the crowd have managed to control the pandemic.	No smart apps yet
sanction	Anyone who spreads disinformation about the virus is also subject to punishment.	Punishment for spreading hoaxes of Covid

## COVID-19 Risk Perception

The perceived risk of COVID-19 is defined as a measure of how threatened an individual feels about a health problem, the ratio between the benefits of taking a particular action and the barriers to it, and a marker that alerts individuals to certain healthy behaviors. Its level is influenced by perceptions of vulnerability and seriousness. Risk perception is an important issue in today's world, where the COVID-19 pandemic has yet to be resolved. Effective risk management is not enough with only physical programs such as procurement of goods or the development of certain infrastructure, but it is necessary to pay attention to the human aspect so that there is active involvement of the community (Durst et al., 2020). Subjectivity in perceiving risk can make people not aware of the objective risks that will be faced. The term risk refers to the probability of a hazard occurring (Denney, 2005). Knowledge or markers of risk help to avoid danger. In the condition of the COVID-19 pandemic, the psychological response of anxiety or worry emerges as a marker of risk perception. In relation to our research, the perception of risk felt by tourists varies, depending on the amount of experience a person has in making a trip (Fuchs and Reichel, 2011). The higher the risk perceived by an individual, the higher the prevention efforts that will be carried out by the individual (Khosravi, 2020). For instance, the increasing number of deaths resulting from COVID-19 led the public to perceive its risk (Bavel et al., 2020). The number of threats is not necessarily balanced with anticipatory behavior, as found in cases of natural disasters or health risks. An understanding of the community's risk perception is needed, for example about the factors that influence prevention behavior.

### Fear of COVID-19

Fear is an emotion found in humans that appears naturally. The fear arises due to the biochemical response as well as the individual's high emotional response. When there is a threat or danger, fear arises and warns the individual to be careful. The threat or danger can be in the form of physical or non-physical threats According to Olsson and Phelps (2007), fear is an emotion that is formed due to danger or a threatening situation. Ahorsu et al. (2020) explained that the COVID-19 pandemic and its spread caused fear, worry, and anxiety in people around the world. One of the things that characterize infectious diseases with other health conditions is fear. The fear that arises is related to the speed and medium of its spread as well as its comorbidities and mortality. In addition, psychosocial aspects related to fear are the emergence of stigma, loss, and discrimination (Pappas et al., 2009).

Fear of COVID-19 is an unpleasant emotion felt by individuals due to a threat or unusual event, in this case, a disease outbreak or epidemic. There is a fear of being infected with a virus, losing a job, losing loved ones, and various other aspects of life during the COVID-19 pandemic (Pakpour and Griffiths, 2020).

### Fear to Travel

According to Adolph (2013), fear is something that happens suddenly and is felt to be dangerous or threatening. Fear can also be interpreted as an unpleasant feeling that is triggered by the perception of danger, real, or imagined. Physiologically, symptoms of fear or anxiety can take the form of sweaty palms, shaking, dizziness, or heart palpitations when individuals are faced with challenging situations. Based on phenomenology, the expressions of human behavior and emotions because of fear are different from one another (Barlow, 2002). Beck and Emery (1979) define fear as a judgment because a situation is perceived as dangerous. In addition, fear can also be interpreted as an unpleasant emotional state and is triggered by the perception of a threatening design.

Fear can occur because an individual is unable to adapt to his environment, and that fear is also caused by threats that someone can avoid and so on (Gunarsa, 2008). When there is a danger or threat to the individual, then fear will arise and warn the individual to be careful. Individual responses to the results of challenging living conditions, in general, can be in the form of shock, panic, stress, and post-traumatic stress disorder (Aydin, 2020). Nurishaq (2020) explained in his research that the COVID-19 pandemic in addition to destroying the order of life also caused various psychological disorders in the form of stress including fear and anxiety. Moerti (2020) stated that the number of positive cases of COVID-19 was increasing day by day. The increasingly high number of cases of COVID-19 transmission in Indonesia indirectly makes people afraid of contracting the virus and afraid when traveling (Gunagama et al., 2020).

### Risk Perception to Travel

According to Sjöberg et al. (2004), risk perception is an individual's subjective assessment of a particular situation and how much attention the individual pays to the consequences. In addition, Mullai (2006) added that risk perception is the result of interpretation of a person's assessment of risk, whether the risk faced is still tolerable or not. Bhasin (2018) defines risk perception as uncertainty or analyzing potentials that may occur in the future. Perceived risk is a negative consequence that is anticipated by consumers regarding the situation of purchasing a product or service. In addition, risk perception can be defined as a subjective evaluation of the situation that occurs or threatens the individual. Risk is considered to be different and can affect an individual's behavior (Weinstein, 1984). Perception of Risk in tourism can be associated with the evaluation of the situation regarding the risk in deciding on a trip (traveling), buying or consuming products, and travel experiences (Reisinger and Mavondo, 2005). Perception of risk in tourism is related to the possibility of the action of a hazard that can affect travel decisions (Chew and Jahari, 2014). Fuchs and Reichel (2011) suggest that tourists can experience different levels of risk, this depends on the amount of experience a person has in traveling. In addition, the experience of traveling to a certain destination not only affects the intention to visit these places but can also influence a person to avoid areas or places that he thinks are at risk.

Utama and Setiawan (2020) explained that risk perception has four dimensions including financial risk, time risk, social-psychological risk, and health risk. Financial risk is the possibility of tourists losing money or money benefits due to bad decision making. When individuals decide to travel, they have the potential to run out of money when traveling due to the economic uncertainty triggered by the COVID-19 pandemic. Time risk is the possibility that the person traveling considers their time wasted (Quintal et al., 2010). Social-psychological risk in this case is an uncomfortable feeling that arises from anticipated post-behavioral emotions such as worry, anxiety, or tension as well as an individual worries about getting a negative response from the people around them, such as humiliation, making the individual feel ashamed (Mowen and Minor, 2002). Health risk refers to the risk of contracting a disease due to COVID-19. Individuals who feel at risk of contracting a disease or virus will avoid travel to minimize the health risks that occur (Brewer and Fazekas, 2007).

### Vaccine Attitude

Martin and Petrie (2017) attitudes toward vaccines are attitudes toward giving vaccines either individually or in groups. The factors that underlie a person's acceptance/rejection of vaccination are distrust of the application agency, receiving information about vaccines, distrust of crime, and vaccine safety. Zarobkiewicz et al. (2017) explain that there are differences in anti-vaccine attitudes between medical students with other non-medical students. This shows that one of the factors that cause people to have anti-vaccine attitudes is vaccine knowledge. Hornsey et al. (2018) found that the main problems with individuals who have anti-vaccine attitudes are lack of exposure to information and failure to digest information. Anti-vaccine attitudes are not only owned by less educated individuals (Larson et al., 2014). Educated individuals will tend to spend a lot of time searching for information on social media or the internet about vaccination (Jones et al., 2012).

### Travel Intention

Intention is something that is involved with a person's behavior (Oliver, 1997). Meanwhile, according to Ajzen (2005) intention is an antecedent of a visible behavior. Supported by Shen et al. (2009), travel intention is an individual's readiness to enact a behavior and is considered to be directly affected by behavioral antecedents. Conner and Norman (2005) say that intention is a person's behavior representing one's decisions. As in Winarta et al. (2016) intention is an awareness that motivates a person's decision to complete a behavior. The intention is a subjective probability that a person has to perform a certain behavior (Fishbein and Ajzen, 1975).

Travel intention itself comes from the concept of “the intention,” one of the dimensions in the Theory of Planned Behavior theory proposed by Fishbein and Ajzen (1975). Meanwhile, the intention concluded by Ajzen (1991) is to determine the motivational factors that can influence behavior, including an indication of how hard people try, how much effort they plan to exert to carry out the behavior. Travel intention refers to the possibility to visit a certain destination in a certain period and in the future (Whang et al., 2016).

### Research Hypothesis

**Figure 1:**
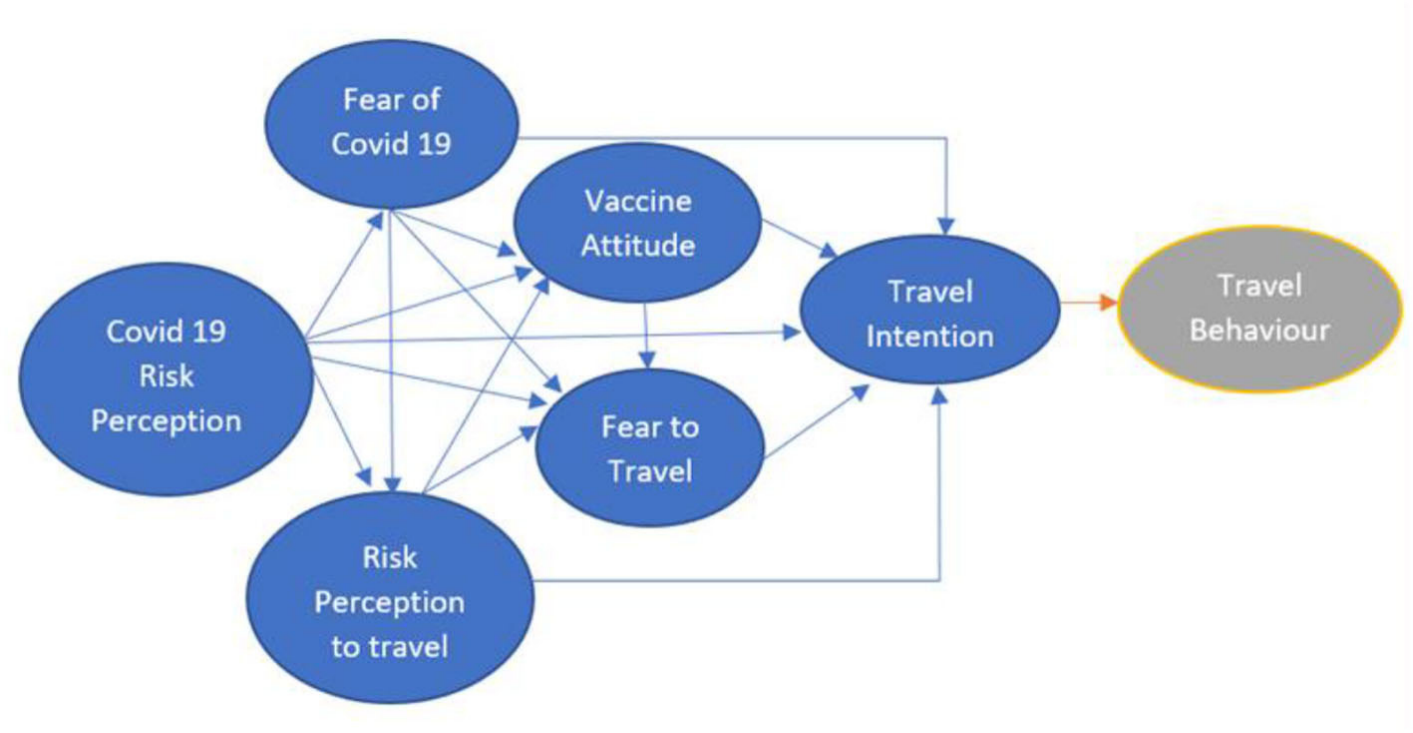
Assumed model.

There is a direct or indirect partial role between COVID-19 Risk Perception, Fear of COVID-19, Risk Perception to Travel, Vaccine Attitude, and Fear of Travel on Travel Intention during the pandemic for Indonesian and Taiwanese tourists as designed in Figure 1.

Participants were selected by using a purposive sampling technique, considering the determination of the sample is based on certain characteristics or criteria which are relevant to this present study. The youth criteria involved in this survey were Indonesian or Taiwanese citizens, at least 18 years of age, affected by the COVID-19 pandemic, and have had or presently have a strong urge to travel. The survey used was a Likert scale because it has several alternative answers from very positive to very negative answers where each choice has a score (Sugiyono, 2015). Respondents were given instructions to select the best answer of each statement which reflected their personal values from a list of scales or replies available on the online survey platform “google forms.”

The study was conducted in Indonesia and Taiwan from September 19, 2021, to October 15, 2021, and obtained 358 Indonesian respondents and 283 Taiwanese respondents. After checking all existing responses, no errors were found in the results of the research scale. Researchers examined the descriptions of research respondents in order to describe their characteristic gender, age, area of origin, last education, and occupation.

## Results

The internal consistency of the questions in which Likert scale used to measure the personal report in our study was reliable, as indicated in Table 2 by the Cronbach's alpha of the six variables were α > 0.6.

**Table 2:** Research scale.

**Scale**	**Dimensions**	**Item**	**Reliability**	**Source**
COVID-19 Risk Perception	perceived susceptibility	6	0.72	(Asefa et al., 2020)
	perceived severity	6	0.68	
Fear of Covid	Emotional fear reactions	4	0.82	(Montazeri et al., 2003; Ahmadzadeh et al., 2013; Ahorsu et al., 2020)
	Symptomatic expressions of fear	3		
Pandemic Anxiety Travel Scale (PATS)	Cognitive	2	0.93	(Zenker et al., 2021)
	Emotional	3		
	Behavioral	3		
Travel Risk Perception	Financial Risk	4	0.70	(Jun, 2020; Utama and Setiawan, 2020)
	Time Risk	3		
	Social Psychological Risk	7		
	Health Risk	5		
Vaccine Attitude (VAX)	Mistrust of vaccine benefits	3	0.94	(Martin and Petrie, 2017; Berman et al., 2020)
	Worries or unforeseen future effects	3		
	Concerns about commercial profiteering	3		
	Preference for natural immunity	3		
Travel Intention	Attitudes	9	0.84	Jehane et al., 2019
	Subjective norms	3		
	Behavioral control	2		

The linearity test was carried out to determine whether or not there was a relationship between variables and whether the relationship showed a straight line or not. The linearity test was carried out by looking for linearity using the statistical program SPSS version 24. The linear assumption test for both Indonesian data and Taiwanese data showed that the linearity results were met as presented in Table 3.

**Table 3:** Two-country partial linearity test results.

**Association**	**Data**		**Information**
	**Indonesia**	**Taiwan**	
Vaccine attitude–travel intention	0.00	0.00	linear
Covid risk perception–travel risk perception	0.00	0.00	linear
Fear to travel–travel intention	0.002	0.00	linear
Fear of covid–travel intention	0.709	-	Not Linear
Fear of covid–fear to travel	0.00	-	linear
Fear of covid–travel risk perception	0.00	-	linear
Fear of covid–covid risk perception	0.00	-	linear
Covid risk perception–vaccine attitude	0.01	0.00	linear
Covid risk perception–fear to travel	0.00	0.00	linear
Covid risk perception–travel intention	0.068	0.437	Not Linear
Travel risk perception–fear to travel	0.00	0.00	Linier
Travel risk perception–travel intention	0.00	0.00	Linier
Vaccine attitude –fear to travel	0.00	0.00	Linier
Vaccine attitude–travel risk perception	0.00	0.00	Linier
Vaccine attitude–fear of covid	0.01	-	Linier

The research was conducted using multiple and simple linear regression methods to test the proposed hypothesis. Table 4 aims to prove the simultaneous effect of independent variables on the dependent variable. The simultaneous effect can be proven through a significant value of less than 0.05.

**Table 4:** Indonesia.

**Model**		**Sum of squares**	**df**	**Mean square**	**F**	**Sig**.
1	Regression	7,877,266	5	1,575,453	20,460	0.000^b^
	Residual	27,105.158	352	77.003		
	Total	34,982.425	357			

^a^*Dependent Variable: Travel Intention*.

b *Predictors: (Constant), Fear To Travel, Anti Vaccine Attitude, Covid Risk Perception, Fear Of Covid, Risk Perception To Travel*.

Based on Table 5, it is shown that the independent variables, namely Fear To Travel, Anti Vaccine Attitude, Covid Risk Perception, Fear Of Covid, and Risk Perception Of Travel can predict the dependent variables, namely travel intention in Indonesian tourists. Table 5 shows that it is simultaneously proven that the variables Fear To Travel, Anti Vaccine Attitude, Covid Risk Perception, and Risk Perception To Travel can predict travel intention in Taiwanese tourists.

**Table 5:** Taiwan.

**Model**		**Sum of Squares**	**df**	**Mean Square**	**F**	**Sig**.
1	Regression	4,848,005	4	1,212,001	15,416	0.000^b^
	Residual	21,856,787	278	78.622		
	Total	26,704.792	282			

^a^*Dependent variable: travel intention*.

b *Predictors: (Constant), fear to travel, vaccine attitude, risk perception Covid, risk perception travel*.

Table 6 is a partial test in Indonesian and Taiwanese research, proven to have a partial relationship between all independent variables such as Fear To Travel, Anti Vaccine Attitude, Covid Risk Perception, Fear Of Covid, and Risk Perception To Travel on the dependent variable, namely travel intention, and this can be seen from the sig value of less than 0.05. Meanwhile, the results of the partial test of the Taiwanese study showed that of the five dependent variables, fear of covid was not proven to form a travel intention model in Taiwan, so only four variables formed Taiwan's travel intention, namely Covid Risk Perception, Risk Perception to Travel, Anti Vaccine Attitude, and Fear to Travel.

**Table 6:** Partial test of the independent variable on the dependent variable.

		**B**		**Sig**	
		**Indonesia**	**Taiwan**	**Indonesia**	**Taiwan**
1	(Constant)	20.147	27,876	0.000	0.000
	Covid risk perception	0.202	0.044	0.014	0.540
	Fear of Covid	0.186		0.041	
	Risk perception to travel	−0.106	−0.079	0.010	0.079
	Anti vaccine attitude	0.439	363	0.000	0.000
	Fear to travel	−0.239	−0.403	0.003	0.000

*Dependent variable: travel intention*.

Based on the table of results of hypothesis testing, Indonesian data shows a significance level or p-value of 0.001 <0.05 (*p* = 0.001), which means that the alternative hypothesis proposed by the researcher is accepted. The table can also explain that there is a correlation or relationship between the independent variable (X) and the dependent variable (Y) with a relationship value of 0.225. The results of the coefficient of determination can also show that all of the independent variables, namely Fear To Travel, Anti Vaccine Attitude, Covid Risk Perception, Fear Of Covid, and Risk Perception To Travel, can predict the dependent variable, namely travel intention with an effect of 22.5%, while 77, the other 5%, is the influence of external variables which are not the focus of this research.

On the other hand, the Taiwan research data shows the results of the hypothesis test of a significance level or p-value of 0.000 <0.05 (*p* = 0.001), which means that the alternative hypothesis proposed by the researcher is accepted. The table can also explain that there is a correlation or relationship between the independent variable and the dependent variable with a relationship value of 0.182. The results of the coefficient of determination can also show that all independent variables namely Fear To Travel, Anti Vaccine Attitude, Covid Risk Perception, and Risk Perception To Travel can predict the dependent variable, namely travel intention with an effect of 18.2%, while the other 81.2% are the influence of external variables that are not the focus of this study.

These values indicate that the regression model can be used to predict the travel intention variable, in other words, there is an effect of the variables Fear to Travel, Anti Vaccine Attitude, Covid Risk Perception, Fear of Covid, Risk Perception to Travel on the travel intention variable (Y). The diagram of the results of testing the Indonesian data hypothesis can be seen in the image below.

**Figure 2:**
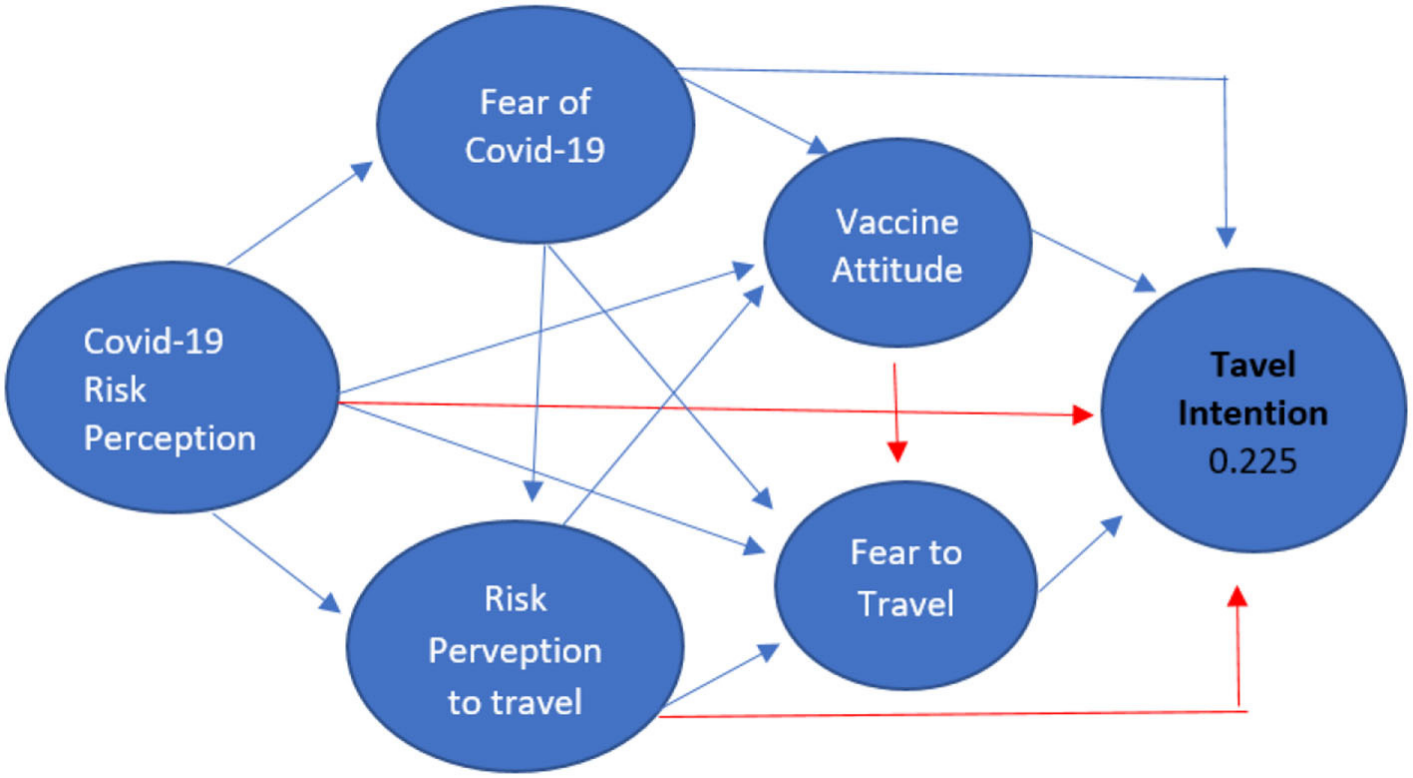
Indonesia travel intention research results.

The model test shows a significant role between Fear of Travel, Anti Vaccine Attitude, Covid Risk Perception, Fear of Covid, and Risk Perception to Travel on the travel intention variable, but the findings of the study show some partial results for each correlation between each variable (see Figure 2). Of the four independent variables, COVID-19 risk perception and risk perception to travel do not have a direct role in travel intention. However, the two variables can play a role in the travel intention variable when calculated simultaneously with the other three variables. In addition to COVID-19, risk perception and risk perception to travel can play an indirect role in travel intention. COVID-19 risk perception can play an indirect role through the variables Fear to Travel, Anti Vaccine Attitude, Fear of Covid, and Risk Perception to Travel.

**Figure 3:**
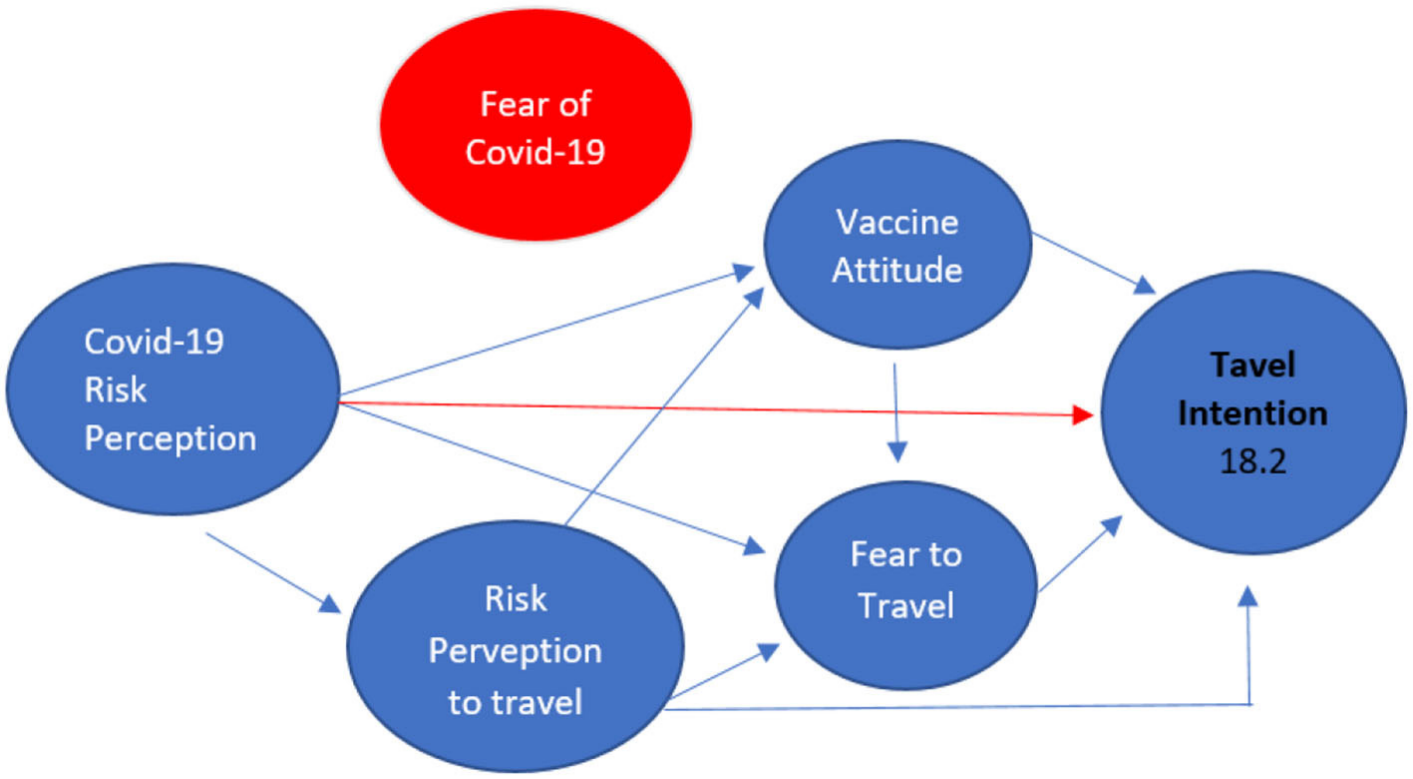
Taiwan travel intention research results.

Different results are shown from the Taiwan data test as illustrated in Figure 3. The Taiwan research shows that the variable fear of covid cannot be included in the theoretical model that builds travel intention. The figure below shows the results of the Taiwan study.

Similar to the results of the Indonesian study, the results of the Taiwanese study show that there is no direct role of Covid Risk Perception on Travel Intention, but the indirect role of Covid risk perception on travel Intention can occur through Travel Risk perception, Fear to travel, and vaccine attitude.

## Discussion

Indonesian respondents totalled 358, while Taiwanese respondents amounted to 283. Each of the research respondents and regions—Indonesia and Taiwan—had their own discussion. Our research has shown that during the pandemic, Indonesian travel intentions were predicted by COVID-19 risk perception, fear of COVID-19, risk perception to travel, attitude toward vaccination, and Fear of Travel. In Taiwan, however, outcomes of Travel Intention are influenced by COVID-19 Risk Perception, Risk Perception to Travel, Vaccine Attitude, and Fear to Travel. It's interesting to note that among Taiwanese travel intentions were not significantly correlated with Fear of COVID-19.

The results of testing the partial role of Indonesian research directly or indirectly proved significant between COVID-19 Risk Perception, Fear of COVID-19, Risk Perception to Travel, Vaccine Attitude, and Fear of Travel on Travel Intention during a pandemic. However, the direct role of risk perception, both covid risk perception and travel risk perception, on travel intention is not proven. Covid risk perception and travel risk perception are only proven through intermediary variables such as fear of covid, fear of travel, and vaccine attitude. Taiwanese respondents proved a partial role between Vaccine attitude and Fear of Travel.

Travel Intention in Indonesia is built by covid risk perception, travel risk perception, fear of covid, vaccine attitude, and fear to travel. The five independent variables simultaneously play a role in shaping travel intention. Although the pandemic period has had a major impact on the tourism industry, humans still need tourism activities amidst the limitations and disasters of the pandemic. Farmaki (2021) states that there is a process of forgetting about crises such as a pandemic in tourism behavior. Even though the pandemic has had a bad impact on the tourism industry after the crisis has passed, people tend to forget the crisis and return to tourist behavior quickly. It becomes a separate question for the formation of travel intentions. Several studies state that travel intention is shaped by risk perception, Reisinger and Mavondo (2005); Li and Ito (2021); Sujood and Bano (2021) explained that covid risk perception affects travel intention. However, Luo and Lam (2020) through their research results state that covid risk perception cannot directly affect travel intention, but has an indirect affect through fear of travel. As mentioned, fear of travel can play a direct role in travel intention and will be stronger when a traveler has high self-efficacy (Klabi, 2021). The same thing is explained by the research of Luo and Lam (2020); Reisinger and Mavondo (2005), Zheng et al. (2021) found that fear to travel has a direct role in travel intention. Another form of travel intention is vaccine attitude, according to Gursoy et al. (2021), Poulos et al. (2018) vaccine attitude can affect travel intention.

Of the five independent variables, only vaccine attitude and fear to travel have a direct role on travel intention without the role of intermediary variables. Research conducted by Martin and Petrie (2017) found that there are several forming factors of vaccine attitude that can be identified, namely vaccine behavior intention, sensitivity to medicines, current health, and media. This study also found that there are four dimensions of vaccine attitude, namely mistrust of vaccine benefits, worries about unforeseen future effects, concerns about commercial profiteering, and preference for natural immunity. Mass media coverage of vaccines has a positive and significant impact on the knowledge of the consequences of vaccines and intentions to vaccinate before traveling abroad. Research conducted by Radic et al. (2021) further explains that the mass media can convey the effectiveness of vaccines, provide accurate information, and increase public knowledge about the COVID-19 vaccine program. This is so that the message conveyed by the mass media is built on people's hopes and enthusiasm for the COVID-19 vaccine and has a positive impact on people's intentions to vaccinate before traveling abroad. Research conducted by Zheng et al. (2021) found that fear to travel has a negative relationship with tourist attractions to be visited due to the severity of the pandemic. The results of this study indicate that the severity of threats and vulnerabilities can lead to travel fear which can ultimately affect motivation related to health risk behaviors (smoking, HIV, vaccines) and protective behavior for travel. This is under the research of Zheng et al. (2021) regarding the prediction of travel behavior in the post-pandemic community, which found that public fear significantly increases travel avoidance.

Li et al. (2021) mention that global health emergencies evoke three types of tourism patterns: from general to complex, from open to closed, and from radical to conservative. These categories provide a conceptual basis for empirical research, taking into account contextual and individual stimuli. Practically speaking, this paper highlights strategies for reducing individual risk perceptions and encouraging certain types of tourism. The recommendations also encourage crisis recovery analysis and relevant market analysis by tourism professionals and marketers. Risk perception is said to have a big role in the formation of travel intention. The results of this study indicate that risk perception, neither covid risk perception nor travel risk perception has been shown to have a direct role on travel intention. However, the two independent variables can influence the travel intention variable through other intermediary variables such as fear of covid, vaccine attitude, and fear to travel. Following the results of this study, the perception of risk alone is not enough to influence travel intention, this is confirmed by the results of research by Qiu et al. (2020) that residents perceive the risks posed by tourism activities, and estimate their willingness to pay to reduce public health risk based on a hypothetical scenario, using a triple-bounded dichotomous choice contingent assessment method. Social costs in the three urban destinations were assessed and compared. Based on the findings, suggestions are made for appropriate post-pandemic recovery actions by local authorities and tourism organizations. Li and Ito's (2021) research on the people of Sapporo and Wuhan found that people in Sapporo's perception of COVID-19 risk negatively affected their travel intention at the start of the pandemic period. However, data in Wuhan shows that although there is a negative influence of perceived COVID-19 risk on travel intention, this influence may be temporary until the restrictions or lockdowns are terminated. This shows that various other factors mediate the covid risk perception of travel intentions.

Variables that have a direct role in travel intention are fear of covid, vaccine attitude, and fear of travel. Research conducted by Klabi (2021) states that anxiety about COVID-19 has a negative influence on intentions to travel by air. Research conducted by Zheng et al. (2021) states that if the high severity and magnitude of the threat from COVID-19 is a factor that can cause a person to experience travel fear, it directs a person to increase motivation toward protection and protective attitude when traveling after a pandemic outbreak. Fennell (2017) explains that the concepts of fear to travel are formed from various factors such as obstacles, shock, panic, risk, anxiety, and worry. This fear to travel will affect travel intention. In addition, media coverage related to the impact of mobility on the severity of the pandemic and vaccinations being promoted has a significant influence on travel intentions. Similarly, the results of research conducted by Zheng et al. (2021) on tourists in China showed that tourists were fearful, as 34.6% of the total respondents stated that there was the highest perceived fear and threat to post-pandemic travel. However, they also showed the highest levels of motivation toward protection and travel avoidance intentions. In addition to fear of both covid and travel, vaccine attitude has been proven to have a role in travel intention. Research conducted (Gursoy et al., 2021) explains the relationship between vaccine intention and travel intention. The initial vaccination program initially resulted in vaccine intention having a negative relationship with travel intention. However, this negative impact subsequently decreased as the number of individuals vaccinated increased significantly, closing the gap between the two groups. The findings in this study also show that socio-demographic factors such as age, gender, marital status, education level, region of residence, race, religion, and occupation affect COVID-19 vaccination intentions and vaccine doubt.

Fear of covid can also convey an indirect message to travel intention through vaccine attitude, travel risk perception, and fear to travel. The results of this study are supported by one of the results of research from Angguni and Lenggogeni (2021), the results of this study reveal that health crises that occur such as the Covid 19 Pandemic can be explained through psychological factors such as anxiety, mediated between tourists' perceptions of travel risks during the Covid 19 period and travel intentions. Several other studies explain that fear of covid can play an indirect role on travel intention through other variables. Research conducted by Luo and Lam (2020) states that fear of covid does not have a direct influence on the desire to travel, however, fear of covid has an indirect influence through travel anxiety and risk attitude as a mediator variable on travel intentions. The overall results of the study state that fear of covid has a significant influence on travel anxiety and risk attitude so it makes a person afraid to travel. In addition, research conducted by Rather (2021) shows that fear of COVID-19 and perceived risk have a significant negative impact on travel attitudes, which makes people afraid to travel.

This research is relevant to the findings of Rahman et al. (2021) that the impact of the COVID-19 pandemic is very large and affects the risk management process, including service delivery, travel patterns, and avoiding tourist destinations that have excess population or visitors. Cleanliness and safety in risky trips are also perceptions that are considered by tourists. Travelers believe that this pandemic creates risky travel and reduces their travel plans. This study also found that travel risk and management perception were closely related to managing risk. Risk management is one of the significant factors that influence individual beliefs regarding how to control threats during a pandemic. On the other hand, tourist behavior can lead to risk management for the destination infrastructure, health facilities, image of the destination, and travel planning. Travel patterns lead to independent or group trips. Nature-based tourists or outdoor activities are also findings that are perceived by the public. This is done to reduce the risk of travel and enable tourists to feel comfortable in traveling during the COVID-19 pandemic.

Covid risk perception can play a role in travel intention only through intermediary variables such as fear of covid, travel risk perception, vaccine attitude, and fear of travel. In a different pandemic context, the research of Cahyanto et al. (2016) examines the factors influencing Americans' avoidance of domestic travel due to confirmed cases of Ebola in the United States in late 2014. The Health Belief Model serves as a theoretical framework for research showing that risk perception is not the only variable that plays a role in travel intention, some results found that perceived vulnerability and self-efficacy significantly influence domestic travel avoidance. These findings also support the significant role of perceived risk, subjective knowledge, age, and gender. Given the possibility that Ebola outbreaks could re-emerge in the future and the emergence of additional health-related crises (e.g., the Zika virus), these findings could also assist the tourism industry in planning and responding to other health pandemics. Most respondents take Ebola seriously and will take protective measures in response to the outbreak. The adapted Health Belief Model helps us understand this phenomenon. As predicted by the model, those with higher risk perceptions, perceived vulnerability, and subjective knowledge were found to be more likely to avoid domestic travel, while those with higher levels of self-efficacy showed lower tendencies to avoid travel due to Ebola. Similar to the covid pandemic, many variables influence the covid risk perception of travel intention, such as fear of covid (Luo and Lam, 2020; Klabi, 2021; Rather, 2021), travel risk perception (Han et al., 2021; Rahman et al., 2021), vaccine attitude (Martin and Petrie, 2017; El-Elimat et al., 2021; Gursoy et al., 2021; Radic et al., 2021), and fear to travel (Reisinger and Mavondo, 2005; Luo and Lam, 2020; Zheng et al., 2021).

The results of research conducted in Hong Kong show that Hong Kong residents are increasingly aware of safety in travel. This supports an increase in intention to travel even though there is a fear of COVID-19 and is causing anxiety in marriages, risk perception harms travel intention, but there is an indirect relationship between fear of COVID-19 and travel intention. Risk and safety are the main things that tourists pay attention to. When anxiety and attitudes toward risk decrease, the intention to travel will increase. In a previous study, it was explained that travel anxiety and risky attitudes were mediating variables between the fear of COVID-19 and the intention to travel. On the other hand, the fear of COVID-19 positively affects travel anxiety, and anxiety risk attitudes also positively affect risk perception. The impact of travel anxiety and risk perception on travel intention is negative. On the other hand, the fear of COVID-19 on travel intention is not significant. This proves that there is no evidence to show that fear of disease reduces an individual's intention to travel.

Travel risk perception can play a role in travel intention if it is through the variable vaccine attitude or fear to travel. The COVID-19 pandemic can lead to a perception of travel risk that can affect travel intention, but in the research of Angguni and Lenggogeni (2021), it is explained that there are other intermediary variables such as fear to travel. Furthermore, Fennell (2017) explains the identification and better understanding of factors and conditions related to travel fear. A literature review of concepts such as constraint, shock, panic, risk, anxiety, and worry formulates the Travel Fear Model. Meanwhile, research by Ruiz and Bell (2021) explains that demographic characteristics, knowledge of vaccines, perceptions of susceptibility to COVID-19, risk factors for COVID-19, and politics may contribute to vaccination doubts and will indirectly influence travel intentions. In the research of Poulos et al. (2018), it is explained that tourists are not unaware of the risks of traveling during a pandemic, they choose to vaccinate before traveling and bear the risk of traveling during a pandemic. In a study conducted in Italy, a group that does not believe in vaccines affects perceptions of risk, including influencing travel behavior (Williams et al., 2020). Other findings also show that the perception of vaccines related to efficacy and protection is also correlated with the risk of travel during a pandemic (Qiao et al., 2021). In addition, the level of trust in vaccines is also influenced by age, gender, and income, where men who are older and have higher incomes tend to believe in the vaccine (Danis et al., 2010). In making travel plans, tourists tend to prefer local locations due to lower perceived risks, and increasing confidence in vaccines will make them willing to travel more broadly in this case regionally (Moya Calderón et al., 2021).

In contrast to the theoretical model of Indonesian travel intention, which has been proven to be supported by all independent variables assumed by the researcher, the results of the Taiwan study show that travel intention is only formed by four independent variables, namely covid risk perception, travel risk perception, vaccine attitude and fear to travel. The fear of covid variable was proven to be unable to be included in the theoretical model of travel intention. The geographical location of Taiwan is quite different from Indonesia, where Taiwan is a country with one large island so policies and supervision related to the procedures for handling COVID-19 can be carried out more effectively and efficiently. The COVID-19 pandemic affected several characteristics such as Taiwan's tourism consumption despite a decline in the frequency of domestic travel, length of travel, and spending during the pandemic, no difference was found between domestic travel expenses and travel plans before and after the pandemic. Travel using public transport for tourism is lower than it was before the outbreak. This is relevant to the results of the study that the fear of traveling does not occur, but most tourists prefer to shorten travel time during the pandemic and are not willing to use public transportation (Li et al., 2021). What is more influential in making decisions to travel is sanitation, the health system in tourist destinations is one of the influential factors (Ivanova et al., 2021). Travel using public transport for tourism is lower than it was before the outbreak.

Although it has a partial role with other variables, covid risk perception is not proven to have a partial role on travel intention. Covid risk perception can affect travel intention through intermediary variables such as travel risk perception, vaccine attitude, and fear to travel. The theoretical model of Travel Intention Indonesia is built by covid risk perception, travel risk perception, fear of covid, vaccine attitude, and fear to travel. The five independent variables simultaneously play a role in shaping travel intention. Although the pandemic period has had a major impact on the tourism industry, humans still need tourism activities amidst the limitations and disasters of the pandemic. Farmaki (2021) states that there is a process of forgetting about crises such as a pandemic in tourism behavior. Even though the pandemic has had a bad impact on the tourism industry after the crisis has passed, people tend to forget the crisis and return to tourist behavior quickly. It becomes a separate question for the formation of travel intentions. Several studies state that travel intention is shaped by risk perception, Bae and Chang (2020); Li and Ito (2021); Sujood and Bano (2021) explained that covid risk perception affects travel intention, but Reisinger and Marvando explained that it is travel risk perception that shapes travel intention. However, Luo and Lam (2020) through their research results state that covid risk perception cannot directly affect travel intention, but must be through fear of travel. As mentioned, Fear of travel can play a direct role in travel intention and will be stronger when a traveler has high self-efficacy (Klabi, 2021). The same thing is explained by the research of Luo and Lam (2020); Reisinger and Mavondo (2005); and Zhang et al. (2020) who found that fear to travel has a direct role in travel intention. Another aspect of travel intention is vaccine attitude, as according to Gursoy et al. (2021), Poulos et al. (2018), vaccine attitude can affect travel intention.

Different from the results of the Indonesian study, travel risk perception has been shown to play a direct or indirect role in travel intention. The indirect role of travel risk perception is through vaccine attitude and fear to travel. Of the five independent variables, vaccine attitude, and fear to travel only have a direct role on travel intention without the role of intermediary variables. Research conducted by Martin and Petrie (2017) found that there are several forming factors of vaccine attitude that can be identified, namely vaccine behavior intention, sensitivity to medicines, current health, and media. This study also found that there are four dimensions of vaccine attitude, namely mistrust of vaccine benefits, worries about unforeseen future effects, concerns about commercial profiteering, and preference for natural immunity. Mass media coverage of vaccines has a positive and significant impact on knowledge of the consequences of vaccines and intentions to vaccinate before traveling abroad. Research conducted by Radic et al. (2021), further explains that the mass media can convey the effectiveness of vaccines, provide accurate information, and increase public knowledge about the COVID-19 vaccine program, so that the message conveyed by the mass media is built on people's hopes and enthusiasm for the COVID-19 vaccine and has a positive impact on people's intentions to vaccinate before traveling abroad. Research conducted by Zheng et al. (2021), found that fear to travel has a negative relationship with tourist attractions to be visited due to the severity of the pandemic. The results of this study indicate that the severity of threats and vulnerabilities can lead to travel fear which can ultimately affect motivation related to health risk behaviors (smoking, HIV, vaccines) and protective behavior for travel. This is following the research of Zheng et al. (2021) regarding the prediction of travel behavior in the post-pandemic community, which found that public fear significantly increases travel avoidance. The results of this study indicate that the severity of threats and vulnerabilities can lead to travel fear which can ultimately affect motivation related to health risk behaviors (smoking, HIV, vaccines) and protective behavior for travel.

This is the same as the results of the vaccine attitude, in contrast to the results of Indonesia in the Taiwan study, the vaccine attitude not only has a direct role on travel intention but also has an indirect role through fear to travel. Li et al. (2020) mention that global health emergencies evoke three types of tourism patterns: from general to complex, from open to closed, and from radical to conservative. These categories provide a conceptual basis for empirical research taking into account contextual and individual stimuli. Practically speaking, this paper highlights strategies for reducing individual risk perceptions and encouraging certain types of tourism. The recommendations also encourage crisis recovery analysis and relevant market analysis by tourism professionals and marketers. Risk perception is said to have a big role in the formation of travel intention. The results of this study indicate that risk perception, both covid risk perception, and travel risk perception, is not proven to have a direct role on travel intention. However, the two independent variables can influence the travel intention variable through other intermediary variables such as fear of covid, vaccine attitude, and fear to travel. Under the results of this study, the perception of risk alone is not enough to influence travel intention, this is confirmed by the results of Qiu et al. (2020) that residents perceive the risks posed by tourism posed by tourism activities, and estimate their willingness to pay to reduce public health risks based on hypothetical scenarios, using the triple-bounded dichotomous choice contingent assessment method. Social costs in the three urban destinations were assessed and compared. Based on the findings, suggestions are made for appropriate post-pandemic recovery actions by local authorities and tourism organizations. Li and Ito's (2021) research on the people of Sapporo and Wuhan found that people in Sapporo's perception of COVID-19 risk negatively affected their travel intention at the start of the pandemic period. However, data in Wuhan shows that although there is a negative influence of perceived COVID-19 risk on travel intention, this influence may be temporary until the restrictions or lockdowns are terminated. This shows that various other factors mediate the covid risk perception of travel intentions. Social costs in the three urban destinations were assessed and compared. Based on the findings, suggestions are made for appropriate post-pandemic recovery actions by local authorities and tourism organizations. Li and Ito's (2021) research on the people of Sapporo and Wuhan found that people in Sapporo's perception of COVID-19 risk negatively affected their travel intention at the start of the pandemic period. However, data in Wuhan shows that although there is a negative influence of perceived COVID-19 risk on travel intention, this influence may be temporary until the restrictions or lockdowns are terminated. This shows that various other factors mediate the covid risk perception of travel intentions. Social costs in the three urban destinations were assessed and compared. Based on the findings, suggestions are made for appropriate post-pandemic recovery actions by local authorities and tourism organizations. Li and Ito's (2021) research on the people of Sapporo and Wuhan found that people in Sapporo's perception of COVID-19 risk negatively affected their travel intention at the start of the pandemic period. However, data in Wuhan shows that although there is a negative influence of perceived COVID-19 risk on travel intention, this influence may be temporary until the restrictions or lockdowns are terminated. This shows that various other factors mediate the covid risk perception of travel intentions. Suggestions are made for appropriate post-pandemic recovery actions by local authorities and tourism organizations.

Meanwhile, fear of travel only has a direct influence on travel intention. Variables that have a direct role in travel intention are fear of covid, vaccine attitude, and fear of travel. Research conducted by Klabi (2021) states that anxiety about COVID-19 has a negative influence on intentions to travel by air. Research conducted by Zheng et al. (2021) states that if the high severity and magnitude of the threat from COVID-19 is a factor that can cause a person to experience travel fear, it directs a person to increase motivation toward protection, and a protective attitude when traveling after a outbreak. Fennell (2017) explains that the concepts of fear to fear to travel are formed from various factors such as obstacles, shock, panic, risk, anxiety, and worry. This fear to travel will affect travel intention. In addition, media coverage related to the impact of mobility on the severity of the pandemic and vaccinations being promoted has a significant influence on travel intentions. Similarly, research conducted by Zheng et al. (2021) on tourists in China showed that fearful tourist as much as 34.6% of the total respondents stated that there was the there was the highest perceived fear and threat to post-pandemic travel. However, they also showed the highest levels of motivation toward protection and travel avoidance intentions. Besides Fear, both covid and travel, vaccine attitude is proven to have a role intention. Research conducted (Gursoy et al., 2021) explains the explains the relationship between vaccine intention and travel intention. The initial vaccination program initially resulted in vaccine intention having a negative relationship with travel intention. However, this negative impact subsequently decreased as the number of individuals vaccinated increased significantly, closing the gap between the two groups. The findings in this study also show that socio-demographic factors such as age, gender, marital status, education level, region of residence, race, religion, and occupation affect COVID-19 vaccination intentions and vaccine doubt. The initial vaccination program initially resulted in vaccine intention having a negative relationship with travel intention. However, this negative impact subsequently decreased as the number of individuals vaccinated increased significantly, closing the gap between the two groups.

Although it has a partial role with other variables, covid risk perception is not proven to have a partial role on travel intention. Covid risk perception can affect travel intention through intermediary variables such as travel risk perception, vaccine attitude, and fear to travel. The findings of this study are in line with research conducted by Zhu and Deng from Sichuan University, who said that knowing the risk of contracting COVID-19 has the potential to harm the intensity of domestic tourist travel (Zhu and Deng, 2020). Furthermore, the research conducted to examine the effect of risk knowledge on travel intention in rural tourism during the pandemic in China explains that this is influenced by risk perception and risk aversion attitude which must be considered together in travel intention activities. This risk perception also affects the number of public transportation users in Taiwan (Kuo, 2021), which causes Taiwanese people to choose to travel short distances instead of long distances (Yang et al., 2021). Moreover, the COVID-19 pandemic affects the level of anxiety when traveling (Zenker et al., 2021), so this fear to travel is generated from the anxiety of traveling that arises from mixed feelings of anticipation, the desire to leave, the unknown, and the fear of leaving a safe home.

Different from the results of the Indonesian study, in Taiwan, travel risk perception has been shown to play a direct or indirect role in travel intention. The indirect role of travel risk perception is through vaccine attitude and fear to travel. Psycho-social risk, such as being afraid of being infected with COVID-19, is an important factor in the lack of confidence in deciding to travel, and this can change if it is known that the health risk reduction while traveling is due to having received the vaccine (Williams et al., 2021). Belief in information on reduced health risks after the vaccine is a triggering factor for voluntary willingness to get the vaccine. Citing the results of his research on people in Italy who were involved in tourism activities. Although this is contrary to the research conducted by Ram et.al., using a three-time cross-sectional study using an online survey (LINE and Facebook) Israeli society stated that the factor for continuing to travel influenced the desire to travel not because it had gotten the vaccine (Ram et al., 2021). In general, the results showed that risk perception accounted for the biggest factor in predicting travel intention among Taiwanese (Sang-Hee et al., 2018; Falahuddin et al., 2020). Regarding Taiwanese society, research by Wong et al. (2021) emphasized that risk perception and fear to travel affect travel intentions abroad and the choice to travel domestically which takes relatively less time. Furthermore, only about 5% stated that they wanted to go abroad for vacations and work assignments, but as many as 48% planned to travel domestically for vacations. Vaccines play an important role in the intention to travel to another country, as this can increase confidence and reduce worries about international travel, such as concerns about time, cost, vulnerability, access, and resilience (Wang et al., 2021).

Similar results were found on the topic of vaccine attitude. In contrast to the results of Indonesia, in the Taiwan study, vaccine attitude not only had a direct role on travel intention but also had an indirect role through fear to travel. Taiwanese tourists who do not travel independently or do not use the services of a travel agent tend to travel for a long time and for a higher number of days (Yang et al., 2021), and during the pandemic, the tourist destinations and tourism plans of the Taiwanese community turned into conservative tourism within the country (Kuo, 2021). In addition, after the pandemic, the number of tourists going abroad decreased because people reduced travel plans that took multiple days. Kuo (2021) added that the level of confidence of the Taiwanese people to travel takes into account state policies related to border control and pandemic prevention, one of which is vaccine services for all people in the territory of Taiwan. As stated in previous research, Taiwanese respondents reduced the desire to travel long distances during the pandemic, unless the country had good policies for prevention, one of which is the administration of vaccines (Ram et al., 2021). This opinion is in line with the research conducted by Wang et al. (2021) who concluded that the desire to travel long distances or abroad had an impact on the high desire to get the vaccine. In another form, it is also conveyed in research that examines the Protection Motivation Theory (PMT) and the role of the government regarding the travel intention of the millennial group. It was stated in the results of this research that state policies during the pandemic to reduce the fear of the risk of contracting COVID-19 had a partial role obtained from the self-efficacy and response efficacy of the Millennial group in their intention to travel (Gengeswari et al., 2021; Williams et al., 2021). In the end, vaccines are one of the most important factors in strengthening self-confidence for travel intention. For this reason, government intervention related to vaccines to increase public protection contributes to travel intention.

Meanwhile, fear of travel has a direct influence on travel intention. In marketing, word of mouth is one of the forces that can influence consumer decisions, which also applies in the world of tourism. As stated by Badreldin and Elbaza (2016) and Filieri and McLeay (2014), there is a change in consumer behavior in the tourism business when receiving scary news, some of which is obtained through eWOM on social media or news available on the internet (blogs, Twitter, and WhatsApp groups) which have become some of the most trusted media to find information for consumers to consider when deciding to travel. Furthermore, in a study in the context of Egypt, travel intention to visit Egypt was strongly influenced by messages of political instability in this country which are exposed to the media causing fear to travel to increase. This is because the media have a significant influence on the perception of the risk of traveling (Scrima et al., 2022). Currently, people have easy access to information because of information technology, which results in the process of obtaining information and knowledge from sources (travel influencers, peer groups, significant others, media) which can have an impact on the fear of traveling which affects travel intention. The results of a case study conducted in three countries in Europe (Germany, Austria, Switzerland) show that Risk Perception and Travel behavior during COVID-19 have an impact on travel intention, this is not only a decrease in the frequency of travel outside Europe but also in countries within the EU mainland (Neuburger and Egger, 2021) and also influences travel intention in Portugal (Magano et al., 2021).

The tourism industry has a vulnerability to crises, and crises have long-term effects on travel patterns, tourist demand, and destination image (Rittichainuwat and Chakraborty, 2009; Chew and Jahari, 2014; Cró and Martins, 2017; Rossell et al., 2017). This is a note of the importance of resilience-based crisis management strategies in the tourism industry (Paraskevas and Quek, 2019). Seçilmi et al. (2021) according to his research, travel influencers (IT) can have a different impact on cognitive reactions and beliefs, which in turn can have an impact on visit intentions, even in pandemic situations. This study aids influencer agencies and the travel industry in developing marketing communications plans to increase travel using social media. In particular, the existing literature recognizes that tourists' perceptions and attitudes of risk toward destinations are heavily influenced by crises, leading to changes in travel plans by avoiding visits to certain destinations or not traveling at all (Sönmez and Graefe, 1998; Lutz and Lutz, 2020). However, in disaster conditions, the intention to travel may decrease but not completely disappear. There are several factors that can cause demand to end, including access changes, environmental hazards, health issues, political unrest, and concerns about the safety of tourists (Morakabati et al., 2012).

Global health emergencies evoke three types of tourism patterns: from general to complex, from open to closed, and from radical to conservative (Li et al., 2020). These categories provide a conceptual basis for empirical research taking into account contextual and individual stimuli. Strategies are needed to reduce individual risk perceptions, encouraging certain types of tourism. The recommendations also encourage crisis recovery analysis and relevant market analysis by tourism professionals and marketers.

## Conclusion, Limitation, and Future Direction

In conclusion, this study indicated that Travel Intention during the Indonesian pandemic was built from COVID-19 Risk Perception, Fear of COVID-19, and Risk Perception to Travel, Vaccine Attitude, and Fear to Travel. Meanwhile, Travel Intention during the Taiwan pandemic was built from COVID-19 Risk Perception, Risk Perception to Travel, Vaccine Attitude, and Fear to Travel. The simultaneous role of Indonesian respondents was proven between COVID-19 Risk Perception, Fear of COVID-19, and Risk Perception to Travel, Vaccine Attitude, and Fear of Travel, on Travel Intention during a pandemic. Taiwanese respondents showed the simultaneous role of COVID-19 Risk Perception, Risk Perception to Travel, Vaccine Attitude, and Fear of Travel on Travel Intention. In the Indonesian research a partial role was found directly or indirectly between COVID-19 Risk Perception, Fear of COVID-19, Risk Perception to Travel, Vaccine Attitude, and Fear of Travel on Travel Intention during a pandemic. However, the direct role of risk perception, both COVID-19 risk perception and travel risk perception, on travel intention is not proven. COVID-19 risk perception and travel risk perception are only proven through intermediary variables such as fear of covid, fear of travel, and vaccine attitude. In Taiwanese respondents, it was proven that there was a partial role between Vaccine Attitude and Fear of Travel on Travel Intention, but there was no direct partial role between COVID-19 Risk Perception and Travel Intention.

This study has certain limitations, for example, firstly we were unable to control the respondents' Social Economic Status (SES), and therefore travel intentions in terms of distances may differ. Second, the transportation systems in Indonesia and Taiwan differ significantly, which influences risk perception in a way that we did not consider. Finally, considering context and culture differences in travel, we would like to analyse gender differences in our study, which is likely to have major variances between Indonesia and Taiwan. We proposed that future studies include the COVID-19 conspiracy theory in order to examine the emotion that contributes to the urge to travel, as media plays an essential role in influencing mental well-being during pandemics, due to individuals receiving information primarily via digital media.

## Ethics Statement

The studies involving human participants were reviewed and approved by the ethics committee of Faculty of Social Science and Political Science, Brawijaya University. The patients/participants provided their written informed consent to participate in this study.

## Author Contributions

All authors listed have made a substantial, direct, and intellectual contribution to the work and approved it for publication.

## Conflict of Interest

The authors declare that the research was conducted in the absence of any commercial or financial relationships that could be construed as a potential conflict of interest.

## Publisher's Note

All claims expressed in this article are solely those of the authors and do not necessarily represent those of their affiliated organizations, or those of the publisher, the editors and the reviewers. Any product that may be evaluated in this article, or claim that may be made by its manufacturer, is not guaranteed or endorsed by the publisher.
